# Tacrolimus-induced parkinsonism in a patient after liver transplantation – case report

**DOI:** 10.1186/s12883-018-1052-1

**Published:** 2018-04-20

**Authors:** Karin Gmitterová, Michal Minár, Miroslav Žigrai, Zuzana Košutzká, Alice Kušnírová, Peter Valkovič

**Affiliations:** 1 Second Department of Neurology, Comenius University, Limbova 5, 833 05 Bratislava, Slovakia; 2 First Department of Internal Medicine, Faculty of Medicine, Slovak Medical University, Bratislava, Slovakia; 3 Institute of Normal and Pathological Physiology, Slovak Academy of Science, Bratislava, Slovakia

**Keywords:** Parkinsonism, Liver transplantation, Immunosupressive therapy, Tacrolimus, Sirolimus

## Abstract

**Background:**

Hepatic encephalopathy may manifest by a wide spectrum of neuropsychiatric symptoms, including cognitive impairment, seizures or extrapyramidal symptoms. The liver transplant can lead to improvement of the signs of encephalopathy but subsequent immunosuppressive treatment might possess pronounced neurotoxicity.

**Case presentation:**

We present a case report of a patient with chronic liver disease who developed signs of Parkinsonism after an orthotopic liver transplant, with consecutive immunosuppressant treatment with tacrolimus. Despite the improvement of liver functions due to the cytostatic treatment, a progressive worsening of neuropsychiatric symptoms associated with the presence of tremor was observed. Metabolic as well as endocrine dysfunctions were excluded as the primary causes of this condition. A brain CT did not reveal structural pathology. Signs of severe, symmetric Parkinsonism - with resting tremor, bradykinesia, rigidity and severe postural instability were observed. A brain MRI was performed with the presence of T2- hyperintensities in basal ganglia bilaterally. Tacrolimus blood concentration was elevated; hence the dose was reduced and later switched to less toxic sirolimus. Subsequently, clinical signs markedly improved after treatment modification. Improvement of clinical symptomatology after tacrolimus discontinuation supports the drug-induced etiology of this neurological condition.

**Conclusions:**

Cytostatic treatment after solid organ transplantation often leads to signs of encephalopathy. If necessary, the dose of cytostatics needs to be reduced, or a less toxic agent must be chosen for the therapy. This modification is usually efficient with no further need for neurological intervention.

## Background

Patients with liver disorders are prone to metabolic encephalopathy – with acute hepatic failure, long-lasting cirrhosis and portal-systemic bypass of circulation, all representing possible causes. These may manifest in a wide range of neuropsychiatric symptoms, including cognitive impairment, confusion, slow speech, loss of fine motor skills, asterixis, peripheral neuropathy, clonus, decerebrate and decorticate posturing, seizures, extrapyramidal symptoms or coma [1]. At the end stage of chronic hepatic disease, only successful liver transplantation can lead to improvement in the signs of encephalopathy. However, this procedure is followed by an immunosuppressive treatment that is well known for its neurotoxicity. We present a case report of a patient with chronic liver disease - without any history of prior neurological complications, who developed signs of Parkinsonism after a liver transplantation with subsequent immunosuppressant treatment.

## Case presentation

The reported patient, a 51-year old woman with biopsy proven primary billiary cirrhosis, was diagnosed in 2000. The only complications present after determining the primary diagnosis were: mild pancytopenia, esophageal varices, portal gastro- and duodenopathy, and splenomegaly. She successfully underwent orthotopic liver transplantation (LT) at the end of January 2016, followed by standard immunosuppressive treatment with mycophenolate mofetil (Cell-cept 500 mg) and corticosteroid treatment (methylprednison 8 mg/day). Due to laboratory signs of early graft rejection (8 days after LT), she was admitted to emergency unit care. After subsequent modification of her previous therapy to continual treatment with tacrolimus (Prograf 5 mg, CZE) (10 mg/day), an improvement in liver function was achieved. Two weeks after the transplant and 7 days of tacrolimus treatment, she developed symptoms of disorientation and confusion. Osmotic demyelination syndrome (ODS) as a consequence of liver transplantation was also considered in the differential diagnosis; however the onset of clinical presentation after LT was delayed as in typical cases. Nevertheless, no supportive signs for this diagnosis were found (e.g. hypo/hypernatremia before LT, relevant peri/post-operative fluctuation of serum sodium/potassium and/or osmotic imbalances) [2]. No surgery related risk factors supportive for ODS (massive bleeding or higher intraoperative fluid intake) were noted either [3]. Thus, metabolic as well as endocrine dysfunctions were ruled out as the major cause of this condition.

**Fig. 1:**
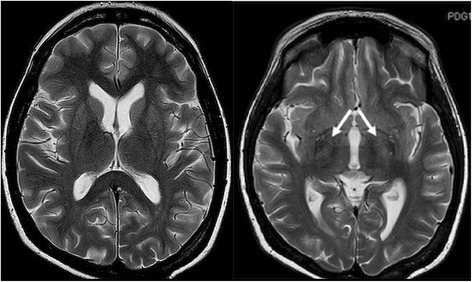
Symmetrical T2-hyperintensity of basal ganglia on MRI (white arrows)

Psychiatric examination was performed pointing to brain dysfunction. The administration of tiapride, thiamine and piracetam was initiated with no resulting benefits. A noncontrast CT brain scan was performed to exclude the structural pathology of the brain with negative result. Because of progressive worsening of consciousness and the development of tremor, neurologists were consulted. In addition to mental disturbances, signs of severe, symmetric Parkinsonism were present - with resting tremor of the chin and all four limbs (MDS-UPDRS III- 4), brady/hypokinesia (MDS-UPDRS III - 3) and rigidity (MDS-UPDRS III –1) [4] revealed by neurological examination. Furthermore, clinical signs of severe postural instability (MDS-UPDRS III -4) were observed. No pyramidal signs or impairment of other systems (oculomotoric, bulbar, cerebellar) were revealed by neurologic assessment. A brain MRI was performed that revealed subtle hyperintensities in T2- sequences in basal ganglia bilaterally without the abnormalities found in the diffusion weighted images (DWI sequences) in the MRI (Fig. 1). Thus, in regard to the chronological course of clinical symptoms related to liver transplantation and subsequent immunosuppressive treatment, we suggested the onset of Parkinsonism was due to tacrolimus-induced encephalopathy. The administration of amantadine, levetiracetam (1000 mg/day) and clonazepam (initial dose 1.5 mg/day) was initiated, with a satisfactory reduction of tremor; and with a positive effect on rigidity and hypokinesia. The tacrolimus blood concentration was above normal levels (21.7 ng/ml, normal value < 20 ng/ml) - the dose was therefore reduced (8 mg/day) and 3 days later, tacrolimus was changed to less toxic sirolimus (Rapamune 1 mg, GBR) according to standard treatment options. One week after the treatment modification and 2 weeks after the first onset of parkinsonian signs, the patient’s tremor and brady/hypokinesia markedly improved (MDS-UPDRS III- 1 and MDS-UPDRS III- 1). No clinically detectable sign of rigidity was found after treatment modification either. Furthermore, no cognitive impairment was observed. Only a mild degree of rest tremor (MDS-UPDRS III- 1) was present in this patient at the time of discharge from the hospital. Due to further improvement of extrapyramidal symptoms, neurological medication was reduced gradually in 3- month follow-up period checks. No clinical deterioration or worsening of the condition was observed during this period. The current treatment includes administration of sirolimus (4 mg/day) with concomitant use of methylprednisolone (2 mg/day) and mycophenolate mofetil (500 mg/day). The sirolimus blood concentration achieved the therapeutic range and no relevant adverse events were observed. No additional treatment of neurological or psychiatric symptoms was needed.

Improvement of clinical symptomatology after tacrolimus discontinuation supports the drug-induced etiology of this neurological condition. It was the subsequent reasoning for not conducting a wide spectrum of differential diagnosing. Subject consent was obtained in agreement with the Declaration of Helsinki and an informed consent from the patient is available.

## Discussion and conclusions

Here we provide a rare extrapyramidal presentation on reversible tacrolimus-induced encephalopathy in a 51-year old woman after liver transplantation. Tacrolimus is a very efficient immunosuppressive drug used especially in solid organ transplantation and despite a wide range of adverse effects tacrolimus still represents the cornerstone of immunosuppressive regimens and the first-line treatment option after liver transplantation [5]. However, approximately 30% of patients experience some form of adverse neurological events [6]. Manifestations vary from minor complaints (e.g. headache) to serious syndromes such as central pontine/extrapontine myelinolysis, speech disorders or neuromuscular complications [7, 8]. Osmotic demyelination syndrome (ODS) represents a severe complication which is usually attributed to a rapid correction of hyponatremia and/or surgery related complications [3]. Symptoms of ODS occur early after surgery and may manifest with various and not rarely atypical course [7, 9]. To date, only few cases reporting the tacrolimus-associated central pontine myelinolysis have been reported in literature [9–12].

Tacrolimus-induced neurotoxicity is usually manifested as encephalopathy, but also a wide range of infectious or cerebrovascular complications might occur [6, 13]. The majority of cases manifest as posterior reversible encephalopathy syndrome (PRES) - with a combination of altered consciousness, seizures, visual abnormalities, headache, vomiting, and/or focal neurological signs [13]. We report Parkinsonism as a dominant sign of encephalopathy induced by tacrolimus. Searching for similar cases described in literature, only one case of Parkinsonism after tacrolimus treatment was reported in a patient with systemic lupus erythematosus [14]. Another study described cyclosporine-induced Parkinsonism in patients after liver transplantation [15]. To date, only one case report of new onset Parkinson Syndrome after LT supporting the diagnosis of tacrolimus-induced Parkinsonism was found in research databases [16].

Even though both substances are classified as immunosuppressive agents; the mechanism acting in the intracellular pathways of sirolimus is different [17]. Tacrolimus reduces the expression of p-glycoprotein in the brain endothelium, leading to dysfunction of the blood**-**brain barrier and vasogenic edema [6] that most likely result in changes to MRI-detectable intensity of various brain regions. Our patient developed neurological signs after transplantation and subsequent immunosuppressive treatment with a laboratory proven liver function improvement. Switching to a less toxic sirolimus led to substantial recovery from all neurological complications. This fact has been previously confirmed [13, 18]. Hence, sirolimus represents an alternative in the occurrence of tacrolimus-induced adverse events and is of special interest for use in hepatic malignancies [5].

Cytostatic treatment - especially one using tacrolimus after solid organ transplantation, often leads to signs of encephalopathy. It may manifest by various neuropsychiatric symptoms, which must be taken into consideration in every patient receiving post-transplantation immunosuppressive treatment. If necessary, the dose of cytostatics needs to be reduced, or a less toxic agent must be chosen for the therapy. This modification is usually efficient, with no further need for neurological intervention.

## Abbreviations

CT: Computer tomography
DWI: Diffusion weighted imaging
FLAIR: Fluid attenuated inversion recovery
MDS-UPDRS: Movement Disorder Society – Unified Parkinson’s Disease Rating Scale
MRI: Magnetic resonance imaging
